# Identification of copy number variation in Tibetan sheep using whole genome resequencing reveals evidence of genomic selection

**DOI:** 10.1186/s12864-023-09672-z

**Published:** 2023-09-19

**Authors:** Huibin Shi, Taotao Li, Manchun Su, Huihui Wang, Qiao Li, Xia Lang, Youji Ma

**Affiliations:** 1 College of Animal Science and Technology, Gansu Agricultural University, Lanzhou, 730070 China; 2 Gansu Key Laboratory of Animal Generational Physiology and Reproductive Regulation, Lanzhou, 730070 China; 3 College of Animal Science & Technology, Henan University of Animal Husbandry and Economy, Zhengzhou, 450046, China; 4 Institute of Animal & Pasture Science and Green Agriculture, Gansu Academy of Agricultural Science, Lanzhou, 730070 China

**Keywords:** Copy number variation, Panou sheep, Whole genome resequencing, Adaptation, Cluster analysis

## Abstract

**Background:**

Copy number variation (CNV) is an important source of structural variation in the mammalian genome. CNV assays present a new method to explore the genomic diversity of environmental adaptations in animals and plants and genes associated with complex traits. In this study, the genome-wide CNV distribution characteristics of 20 Tibetan sheep from two breeds (10 Oula sheep and 10 Panou sheep) were analysed using whole-genome resequencing to investigate the variation in the genomic structure of Tibetan sheep during breeding.

**Results:**

CNVs were detected using CNVnator, and the overlapping regions of CNVs between individual sheep were combined. Among them, a total of 60,429 CNV events were detected between the indigenous sheep breed (Oula) and the synthetic sheep breed (Panou). After merging the overlapping CNVs, 4927 CNV regions (CNVRs) were finally obtained. Of these, 4559 CNVRs were shared by two breeds, and there were 368 differential CNVRs. Deletion events have a higher percentage of occurrences than duplication events. Functional enrichment analysis showed that the shared CNVRs were significantly enriched in 163 GO terms and 62 KEGG pathways, which were mainly associated with organ development, neural regulation, immune regulation, digestion and metabolism. In addition, 140 QTLs overlapped with some of the CNVRs at more than 1 kb, such as average daily gain QTL, body weight QTL, and total lambs born QTL. Many of the CNV-overlapping genes such as *PPP3CA*, *SSTR1* and *FASN*, overlap with the average daily weight gain and carcass weight QTL regions. Moreover, V_ST_ analysis showed that *XIRP2*, *ABCB1*, *CA1*, *ASPA* and *EEF2* differed significantly between the synthetic breed and local sheep breed. The duplication of the *ABCB1* gene may be closely related to adaptation to the plateau environment in Panou sheep, which deserves further study. Additionally, cluster analysis, based on all individuals, showed that the CNV clustering could be divided into two origins, indicating that some Tibetan sheep CNVs are likely to arise independently in different populations and contribute to population differences.

**Conclusions:**

Collectively, we demonstrated the genome-wide distribution characteristics of CNVs in Panou sheep by whole genome resequencing. The results provides a valuable genetic variation resource and help to understand the genetic characteristics of Tibetan sheep. This study also provides useful information for the improvement and breeding of Tibetan sheep in the future.

**Supplementary Information:**

The online version contains supplementary material available at 10.1186/s12864-023-09672-z.

## Background

Copy number variants (CNVs) are a type of genomic polymorphism that may be an important component of phenotypic variation [1]. CNVs are defined as duplications or deletions of genomic segments that range in size from 50 base pairs (bp) to millions of base pairs (Mb) and vary between individuals or species [2]. CNVs have greater effects than single nucleotide polymorphisms (SNPs) and indels in altering gene expression, and they regulate gene expression through gene dosage and positional effects [3]. With the development of sequencing technology and the related advances in molecular biology, the genome has been increasingly understood, and a large number of genetic variants present in the genome have been discovered to have different effects on individual traits. CNVs are an important genetic component of the human genome for many diseases and a major force in human evolution [4]. In the study of human diseases, a large number of genetic diseases were caused by alterations in genome structure. It has been found that CNVs can not only cause major diseases, such as birth defects, but can also be widely distributed in the human genome to cause genetic diversity. CNVs are predicted to be associated with 15% of human genetic diseases; thus, CNVs play an important role in the generation of genetic variation and individual phenotypes in humans [5]. In addition, a wide range of phenotypes and functions in animals and plants are associated with CNVs [6–8]. White fur is more common in sheep, but white wool is easily dyed into other colours. CNVs affect the phenotype of pigmentation and coat colour in domestic animals. The expression level of the agouti-signalling protein (*ASIP*) gene was significantly increased in sheep wool capsules containing a copy of this gene, which was located directly downstream of the *ITCH* promoter [9, 10]. This modified *ASIP* duplication is associated with the typical white coat colour of domesticated sheep breeds. The strong selection signature from the *ASIP* and *KIT* loci in sheep provides conclusive evidence for the strong artificial selection of these duplicated alleles [11]. It was found that the duplication of the *ASIP* gene in goats can also promote white hair colour [9]. Similar to that found in sheep, the coat colour of cattle is mainly determined by the inheritance of different alleles of the *KIT*, *ASIP*, *TYRP1* and *MC1R* genes [12]. Furthermore, studies have found strong evidence that many genes belonging to the innate and adaptive immune systems have variable copy numbers in vertebrate species, particularly in the major histocompatibility complex (*MHC*) genes [13].

It is well known that the evolutionary impact of livestock is due to the environment and selective pressure from tamers. Species diversity arises from the continuous selection of unique external phenotypes by people in the process of repeated breeding [14]. Thus, the external phenotype remains the most obvious result of artificial selection in our domestic species, although this phenotype often escapes simple classification at the molecular level. In recent years, research advances in genomics have resulted in new genotyping tools that allow breeders to identify specific genomic segments to be transferred from parents to offspring for a more precise manual selection of traits. In China, local sheep are divided into three ancestral groups based on their geographical distribution and their genetic origin: Mongolian sheep, Kazakh sheep and Tibetan sheep [15]. With the migration of human beings, Tibetan sheep gradually became the most widely distributed and common livestock on the Tibetan Plateau [16]. Variation in the sheep genome was the basis of widespread phenotypic differences among sheep breeds. Sheep breeds were bred both to adapt them to the local natural environment and to meet human needs. With the long-term presence of natural and artificial selection pressures, different types of domesticated sheep breeds have gradually developed from the wild sheep population [17]. Both the Panou (PO) sheep breed and the Oula (OL) sheep breed are Tibetan sheep, but their individual breeding histories are different. It was reported that Oula sheep were a local breed formed by long-term and continuous hybridization between wild Argali sheep and local Tibetan sheep [18, 19]. Compared with other Tibetan sheep breeds, Oula sheep had unique breed differentiation characteristics [20]. The Panou sheep is an improved breed that was formed after a long period of cross breeding with wild Argali sheep as the male parent and Oula sheep as the female parent using modern genomic polymerization breeding technology [21]. Little research has been reported on the genomic information of Oula sheep and Panou sheep, and there is a gap in research related to CNV in particular.

Currently, the use of whole genome sequencing (WGS) technology to identify CNVs in animal genomes has the advantages of identifying small fragments of CNVs, high coverage, and more accurate results than using aCGH (comparative genomic hybridization arrays) and SNP (single nucleotide polymorphism) arrays [22]. With the dramatic increase in sequencing capacity and the corresponding decrease in sequencing costs, whole genome sequencing data are becoming the primary source of CNV detection. This study aimed to understand the genomic characteristics of Oula sheep (local breed) and Panou sheep (cultivated breed) at the CNV level using a whole genome resequencing approach. This study will deepen the understanding of the physiology and genome characteristics of Panou sheep and provide some theoretical basis for future breeding of Tibetan sheep.

## Materials and methods

### Sample collection and DNA extraction

Blood samples from two populations were collected from the Maqu grassland in the area of Oula Town, Maqu County, Gannan Tibetan Autonomous Prefecture, Gansu Province, China (see Additional file 1: Table S1). The two sheep populations were distributed in different locations in the Maqu grassland, with an average number of more than 300 per population. We randomly selected 10 ewes from each population to collect their blood samples for subsequent genome sequencing. With anaesthesia, 10 ml of blood was collected from the jugular vein of each sheep with a blood collection needle. It was placed in a blood collection tube containing EDTA, stored in liquid nitrogen. The anaesthetic used was procaine, and the instructions for its use were strictly followed during the procedure. Genomic DNA for whole-genome sequencing was extracted from whole blood using a Genomic DNA Purification Kit (Thermo Fisher Scientific Inc., Waltham, MA, USA) [23].

### Library construction and whole genome sequencing

Restriction endonuclease were used for biological digestion of the genomic DNA samples. Then, the repair of the terminus and dA-tail was accomplished by the Paired-End DNA Sample Prep Kit (Illumina Inc., San Diego, CA, USA), which was ligated to the paired-end DNA adaptors. Finally, a 300-bp insert was used for PCR amplification. Genomic libraries were constructed and sequenced using the Illumina HiSeq X Ten (Illumina Inc., San Diego, CA, USA) NGS platform at Genedenovo company (Guangzhou, China) to obtain 150 bp (PE150) paired-end read data. The genome sequencing coverage of all samples was 10×. Raw reads were processed to obtain high-quality clean reads following three stringent filtering standards: (1) reads with ≥ 10% unidentified nucleotides (N) were removed; (2) reads with > 50% bases having Phred quality scores of ≤ 20 were removed; and (3) reads aligned to the barcode adapter were removed [24].

### Sequence alignment and variant calling

The Burrows-Wheeler Aligner [25] software was then used to map the clean reads to the Tibetan sheep reference genome CAU_O.aries_1.0 (https://www.ncbi.nlm.nih.gov/assembly/GCA_017524585.1) with the default parameters. High-quality reads were retained for variant calling by removing multiple aligned reads. Using the Markduplicates function of Picard-2.9.2 software to remove PCR duplicate reads [26]. Genome AnalysisToolkit-3.3.0 was used to realign reads to correct errors caused by InDels.

### CNV and CNVR detection

CNVnator-v0.4 program [27] was used for CNV prediction relative to the CAU_O.aries_1.0 reference assembly, and the optimal bin size was unified to 300 bp for all samples. The CNV calls were filtered with *p value* < 0.01, q0 (zero mapping quality) < 0.5, and size > 1 kb. Estimated copy number per region and copy number between breeds were calculated using the ‘-genotype’ parameter of CNVnator. The ‘CNV_overlap.py’ script on GitHub (https://github.com/bjtrost/TCAG-WGS-CNV-workflow) was used to obtain CNVRs [28]. CNVR could only be used for downstream analyses if it appears in three or more individuals per breed to remove false-positive results [29].

### CNVR annotation

All CNVRs were aligned to the sheep reference genome, and candidate genes were functionally annotated using Gene Ontology (GO) and Kyoto Encyclopedia of Genes and Genomes (KEGG) analysis [30, 31]. GO term categories included molecular function, biological process and cellular component, with a significance level of *p value* < 0.05 for each category. In addition, quantitative trait loci (QTL) of sheep were downloaded (https://www.animalgenome.org/cgi-bin/QTLdb/OA/index) and compared with the identified CNVRs. The Bedtools-v2.27.1 [32] ‘intersect’ command was used to detect the QTLs that overlapped with the identified CNVRs.

### Breed differentiation

Three methods were used to detect breed differentiation based on variation in CNVRs: cluster analysis, V_ST_ statistic and ANOVA test. All sheep individuals were analysed by clustering using the pvcluster R program [33]. Pvclust can perform both clustering and branching point tests, making the clustering results more stable and reliable. In addition, it can provide more detailed statistical information, such as bootstrap confidence and p value of clustering branches, which helps to interpret the reliability and significance of clustering results [34, 35]. A scoring matrix coded as 0 or 1 was used, depending on the presence or absence of CNVRs in each individual. Approximate unbiased (AU) p-values and bootstrap probability (BP) values were calculated by unweighted pair group averaging method (UPGMA) based on the clustering edges shown in red and green, respectively [33]. The highly differentiated regions between the two sheep populations were detected using the V_ST_ statistic method [36]. Only protein-coding genes identified in CNVRs could be used for V_ST_ calculations to study the effects of CNVRs on each breed. We calculated the V_ST_ using V_ST_ = (V_T_-V_S_)/V_T_, where V_T_ is the total variance in copy number between the two sheep breeds and V_S_ is the mean variance within each species [37]. Differences in genes copy number between varieties were calculated using the ANOVA test. Tukey’s HSD test was selected for post-hoc analysis since the sample size was the same for each species (10 samples per species) [26].

## Results

### Identification of CNVs and CNVRs

The sequencing data were mapped to the CAU_O.aries_1.0 reference set, and the read mapping statistics are shown in Table S2 (see Additional file 2: Table S2). The average mapping rate was 99.70%, and the sequencing depth was 10.82×. An average of 1788 deletions and 1232 duplications were detected on the autosomes, with an average deletion size of 20.91 kb and an average duplication size of 19.66 kb (Table 1).

**Table 1:** Statistical summary of detected CNVs

Breed	CNVs	Deletion	Size_Del(bp)	Duplication	Size_Dup(bp)
OL	2998.3	1781.7	20,589.76	1216.6	19899.04
PO	3044.6	1795.2	21,244.19	1249.4	19433.46

**Fig. 1:**
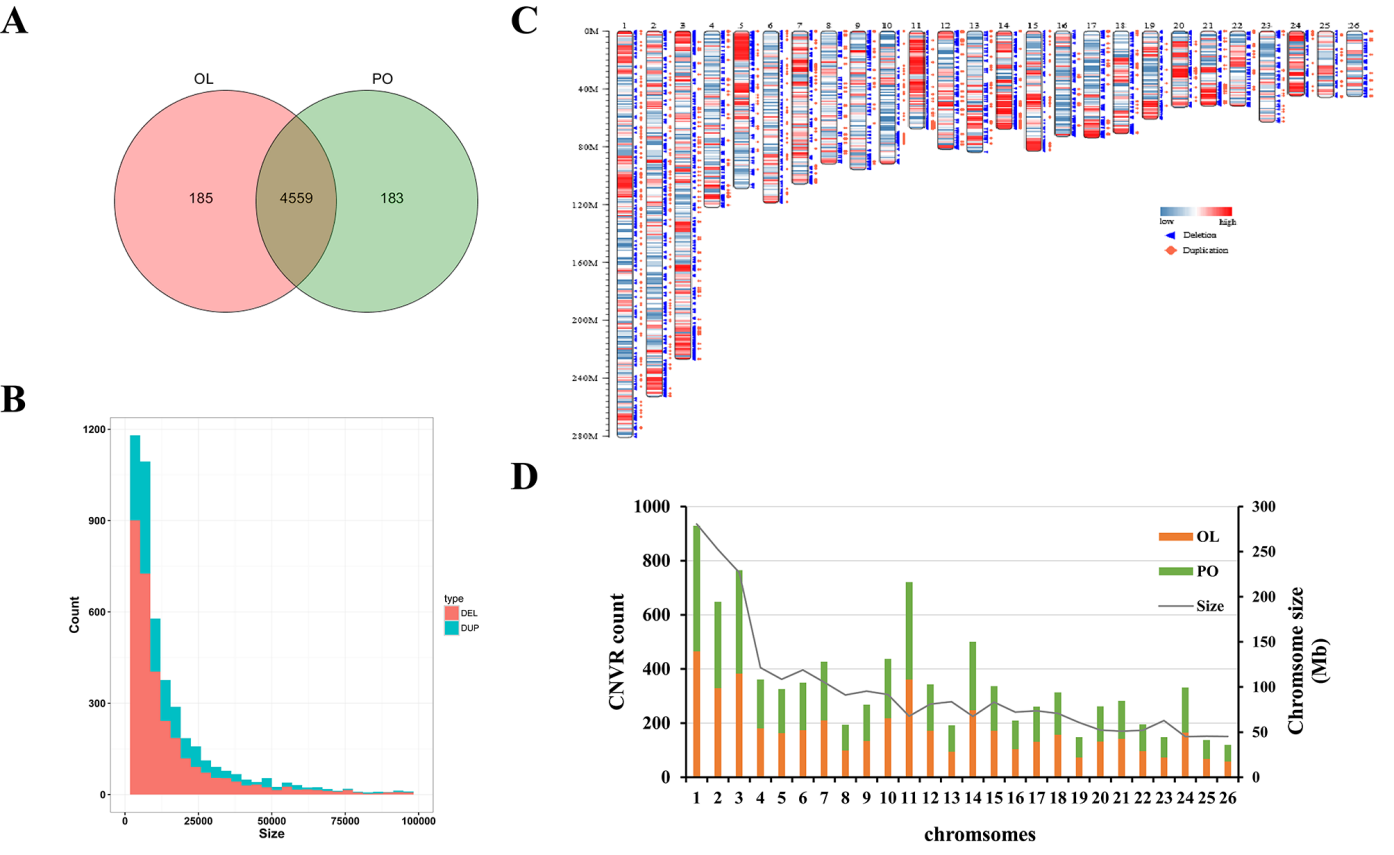
General description of the identified copy number variation regions (CNVRs). **(A)** Venn diagram for overlapping CNVRs between Oula (OL) and Panou (PO) breeds; **(B)** distribution of CNVRs size by state; **(C)** distribution of CNVRs on chromosome ideogram according to their state (deletion and duplication). This was drawn using Rideogram R package. The color painted on the chromosome represents the gene density; **(D)** chromosomal distribution of CNVRs for two Tibetan sheep breeds and length of Tibetan sheep autosome.

We finally obtained 4927 CNVRs in 20 samples. Of these, 3410 were deleted regions ranging in size from 1 kb to 1650.2 kb, and 1517 were duplicated regions ranging in size from 1.3 kb to 368 kb. The numbers of breed-specific CNVRs were 185 (OL) and 183 (PO), (Fig. 1A). Among all loci, OAR 11 had the largest number of CNVRs, with 294 deletions and 188 duplications (see Additional file 3: Table S3). Approximately 3137 deletions were between 1 kb and 50 kb in size, of which 1954 (62.28%) were less than 10 kb. A total of 1377 duplications were between 1.3 kb and 50 kb in size, and 735 (53.37%) were less than 10 kb (Fig. 1B). The Fig. 1C shows the density of genes and distribution of all CNVRs used for analysis. The number of CNVRs on chromosomes did not differ significantly between the two breeds. In general, the number of detected CNVRs was proportional to chromosome size (Fig. 1D).

### Annotation of CNVR

We downloaded the Sheep Quantitative Trait Locus Database (Sheep QTLdb, https://www.animalgenome.org/cgi-bin/QTLdb/OA/index) from the Animal QTLdb database to detect if any identified QTLs associated with CNVRs. A total of 520 QTLs that had been reported in OAR version 3.1 were used for homology comparison (see Additional file 4: Table S4). The results showed that 140 QTLs overlapped with some of the CNVRs by more than 1 kb. Production_QTLs were the most frequently detected, with a total of 26 QTLs overlapping with CNVRs. The most detected Production_QTL was average daily gain, followed by body weight and total lambs born (see Additional file 5: Table S5). A total of 2215 protein-coding genes were annotated in 4927 CNVRs. Approximately 137 CNVRs were annotated to only one gene. At position 3.33 Mb-3.54 Mb in the location of OAR24, up to 27 genes were annotated (see Additional file 6: Table S6).

Functional enrichment analysis of GO (Gene Ontology) terms and KEGG (Kyoto Encyclopedia of Genes and Genomes) pathways was performed by DAVID. Protein-coding genes were enriched in a total of 128 biological processes, 17 cellular components, and 18 molecular functions (Additional file 7: Table S7). In addition, 62 KEGG pathways were significantly enriched (*p value* < 0.05) (Additional file 8: Table S8). The GO terms and KEGG pathways were mainly involved in organ development, neural regulation, immune regulation and digestive metabolism. The GO terms included biological regulation (GO:0065007), regulation of biological processes (GO:0050789), regulation of cellular processes (GO:0050794), developmental processes (GO:0032502) and cellular communication (GO:0007154). Among the KEGG pathway categories, signal transduction (ko04014: Ras signalling pathway, ko04630: Jak-STAT signalling pathway, ko04310: Wnt signalling pathway), skin pigmentation (ko04916: melanogenesis), membrane transport (ko02010: ABC transporters), and digestive system (ko04974: protein digestion and absorption) were significantly enriched.

### Breed differentiation of CNVR

**Fig. 2:**
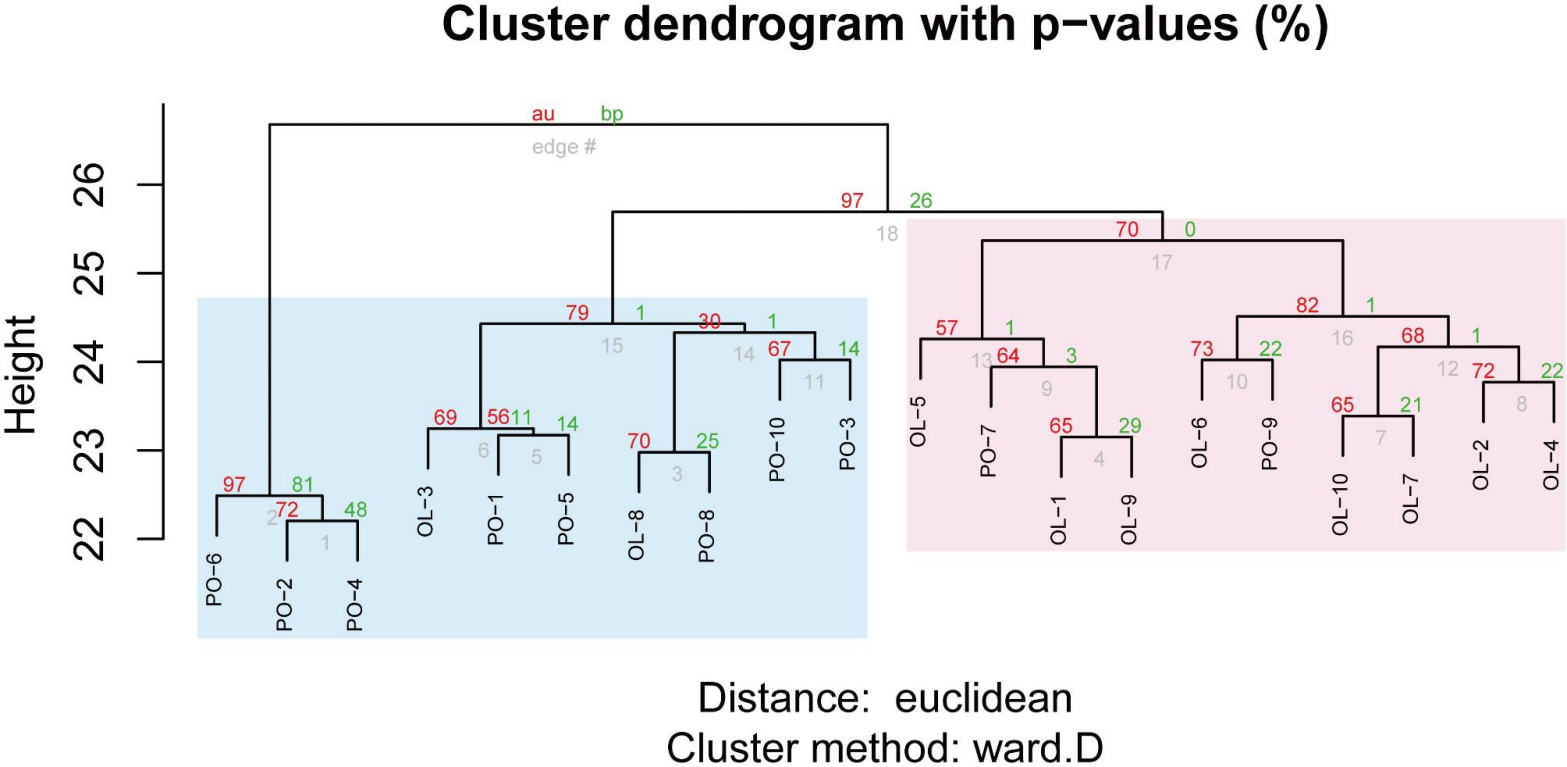
Hierarchical clustering analysis according to the presence or absence of CNVR.

In the two breeds, the hierarchical clustering map was drawn using the scoring matrix to determine whether CNVRs exist in each individual. This matrix indicates the presence or absence of CNVR in an individual and calculates the p value by multiscale bootstrap resampling. The results are shown together with the Approximately Unbiased (AU) p value and Bootstrap Probability (BP) value, and it was confirmed that they were significantly clustered within each breed. The clustering results show that there is some stratification between Panou and Oula sheep (Fig. 2). However, there were some samples that were not well clustered, such as OL-3, OL-8, PO-7, and PO-9. We speculate that OL-3, OL-8, PO-7 and PO-9 belong to heterogeneous individuals present within the group, and their characteristics may be more similar to another group. This phenomenon is quite normal and suggests that the characteristics of individuals are not entirely determined by the group to which they belong but may be influenced by other factors. These factors may include the individual’s genes, environment, etc. [38].

**Fig. 3:**
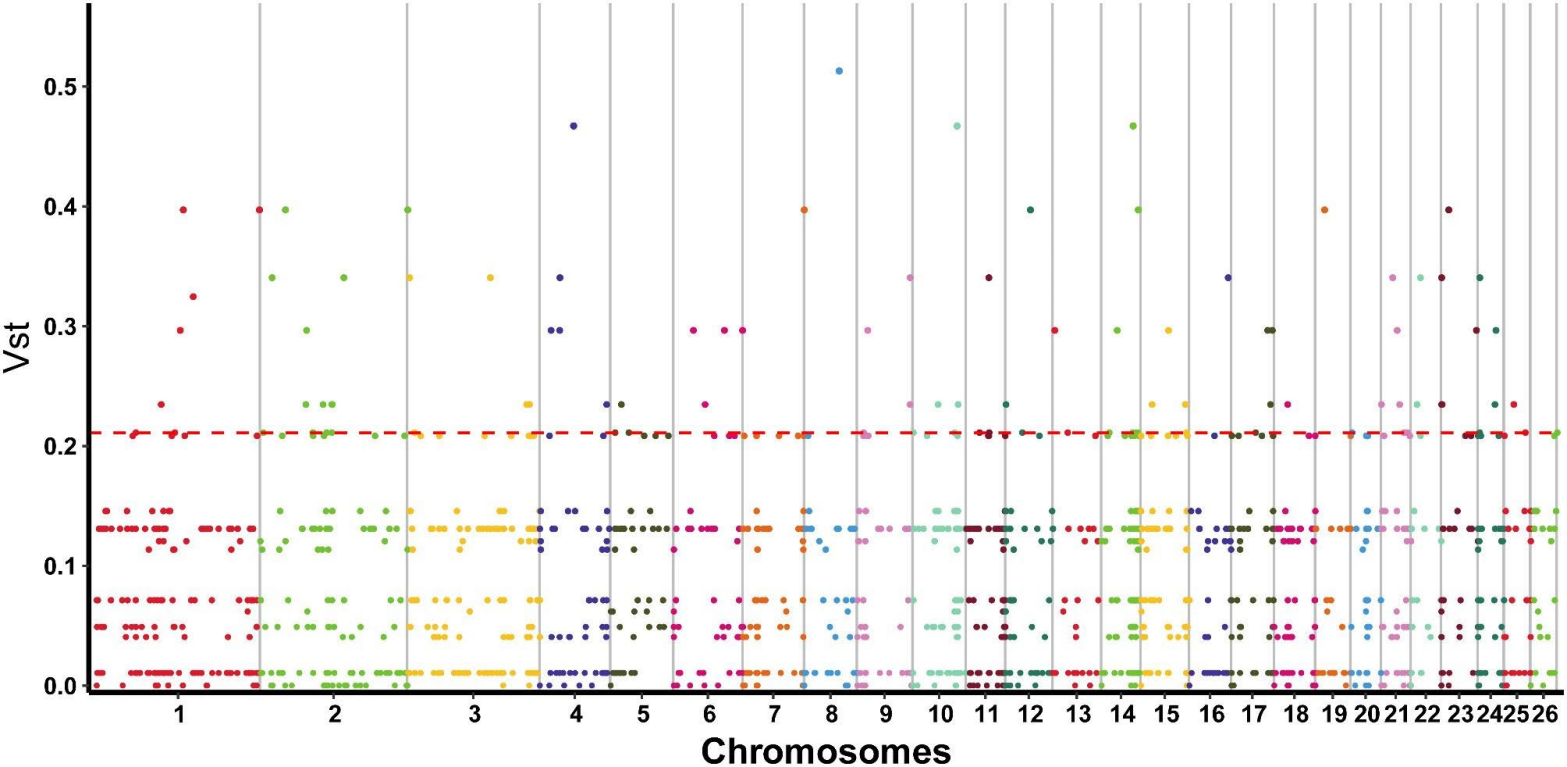
Manhattan plot of genome-wide genes’ V_ST_ value. The red line represents the threshold line

All CNVRs were compared to the sheep genome database, and V_ST_ analysis, ANOVA and Tukey’s test were performed for each CNVR-related gene to identify population-specific selection signatures. The Manhattan plot shows the results of V_ST_, with chromosomes in the horizontal coordinates and V_ST_ values in the vertical coordinates. To observe genes with high differentiation between different breeds, the top 98th [26] percentile was taken as the threshold line (Fig. 3) and genes above this threshold were functionally annotated. The results showed that 41 genes exceeded the threshold in the two breeds (Additional file 9: Table S9). Subsequently, ANOVA and Tukey’s test were used. As a result, 27 genes showed statistically significant differences (*p value* < 0.01) among the varieties (Additional file 10: Table S10). Through functional analysis of the genes above the threshold detection, five candidate genes, *XIRP2*, *ABCB1*, *CA1*, *ASPA* and *EEF2*, related to growth and development, digestion and metabolism, environmental adaptation and reproduction were selected (Table 2).

**Table 2:** CNVR-harboring genes showing high divergence in two population pairs

Chr	Start	End	Type	V_ST_	Symbol	Ensembl Gene ID	Description
2	142,914,601	142,918,000	DEL	0.340278	*XIRP2*	ENSOARG00000004431	xin actin-binding repeat-containing protein 2 isoform X1
4	33,796,501	33,810,400	DUP	0.296296	*ABCB1*	ENSOARG00000001475	PREDICTED: multidrug resistance protein 1 isoform X1
9	90,412,801	90,422,300	DEL	0.340278	*CA1*	ENSOARG00020007556	carbonic anhydrase 2-like protein
11	38,657,201	38,661,500	DEL	0.340278	*ASPA*	ENSOARG00000016806	PREDICTED: aspartoacylase
12	42,276,701	42,279,700	DEL	0.396825	*EEF2*	ENSOARG00000011109	elongation factor 2

## Discussion

Sheep are raised for a variety of purposes, including meat, wool and skin [39]. Sheep can be bred to promote increased productivity, and various forms of variations have been identified in the genome. CNVs affect animal production traits in various ways, such as by deleting genes or altering gene dosage [40]. Among the CNVs identified in this study, “deletion” events were more common than “duplication” events, which is consistent with observations in studies of other species [41]. The results of cluster and V_ST_ analyses showed that there were copy number differences in many genes between the two breeds and that there was some divergence between the Panou and Oula sheep populations. However, the two breeds were relatively close in genetic evolutionary relationships, probably because the two breeds share a common wild lineage ancestor. In our previous PCA results on Oula and Panou sheep, principal components could clearly distinguish Panou sheep from Oula sheep. The minimum number of splits K = 1 in the results of the population structure analysis, indicating that the two breeds are not currently producing a larger population structure. When K = 2 corresponds to our maximum number of clusters, there are two different genetic lineages between the two breeds. Thus, the populations are in a state of low differentiation from each other. When the K value was greater than 2, only four individuals, OL-1, OL-5, PO-2, and PO-6, consistently maintained a separate genetic structural cluster, while all other individuals exhibited more complex genetic structural clusters. Thus, the current artificial and environmental selection pressure is causing the Oula and Panou sheep populations to gradually and continuously diverge in different directions [21].

Genome-wide CNV detection is also a strategy to identify potential key genes for traits of interest by mining genes within CNVRs in a specific experimental design. Even at low coverage, CNV detection using the WGS-based method exhibits better performance than the array-based method [42]. Therefore, we used the different CNVRs between the local breed (Oula sheep) and the cultivated breed (Panou sheep) for the identification of production performance candidate genes to provide a theoretical basis for the subsequent continuous selection of Tibetan sheep. However, due to the possibility of false-positives, we analysed CNVRs for at least three individuals in each breed. We identified 4927 CNVRs in the two breeds, including 3410 deletions and 1517 duplications, and there were a large number of identical CNVRs. This is probably due to their common evolutionary history prior to artificial selection. In this study, the number of CNVRs had a significant positive linear relationship with the length of chromosomes. This result is also consistent with the reports of others [43]. In addition, similar to the results of Huang et al., the CNV assay showed more deletion events than duplication events [44]. Therefore, we speculate that this may be a characteristic of Tibetan sheep. GO enrichment analysis showed that the detected CNVRs overlapping genes common to the two sheep breeds were significantly enriched in organ development, neural regulation, immune regulation and digestive metabolism. The functions related to immune regulation, metabolism and growth and development were also significantly enriched in yak [45] and other sheep breeds [46]. Like the yak, the Oula and Panou sheep live in high altitudes with harsh climates. The strong immune system and the metabolic capacity of the organisms facilitate their adaptation to cold environments with low oxygen at high altitudes and reduce the occurrence of diseases [47]. In the KEGG pathway analysis, it is noteworthy that CNVR-carrying genes common to the two breeds were significantly enriched in digestive metabolism, ABC transporter proteins, disease defence and melanogenesis. In high-altitude and cold regions, the level of nutrient digestion and metabolism is important for Tibetan sheep to maintain homeostasis of their growth and development. This result is similar to that of previous studies on cattle [48] and goats [49]. Studies in mammals have found that ABC transporter proteins can carry a variety of endogenous metabolites through lipid membranes, thereby promoting the absorption and utilization of these nutrients [50]. Thus, the functional genes related to feed digestion and utilization screened in CNVRs may be related to the adaptation of Tibetan sheep to the local living environment.

Many studies have shown that CNVRs contain QTLs associated with economically important traits in animals [51]. Therefore, the CNVRs detected in this study were compared with the QTLs reported in the sheep QTL database. It was found that QTLs affecting economic traits of genetic variation in livestock could be identified by screening the relevant genes contained in genomic CNVs [52]. After integrating the CNVs into the QTLs, 140 QTLs partially overlapped the CNVRs by more than 1 kb. Many overlapping CNV genes, such as *PPP3CA*, *SSTR1* and *FASN*, are located in the average daily weight gain and carcass weight QTL regions. Wan et al. found that the *PPP3CA* gene was involved in the regulation of skeletal muscle growth and development by inhibiting the expression of fibroblast growth factor 23 (*FGF23*). *FGF23* plays a key role in the control of skeletal muscle fibres and is expressed in various muscle tissues [53]. The *PPP3CA* gene was also expressed at high levels in the biceps femoris, longus and abdominal muscles of Tianfu goats, suggesting that the *PPP3CA* gene has an important regulatory role in muscle growth and development [54]. *SSTR1* plays a more important role than other receptors in insulin and GH secretion, especially in the maintenance of basal levels of GH [55]. Li et al. found that the *SSTR1* gene was closely associated with the growth and development of Hulun Buir sheep [56]. The fatty acid synthase (*FASN*) gene is used to improve fatty acid composition [57], and *FASN* is also considered to be an important gene affecting carcass traits in sheep [58, 59]. Therefore, in this study, the candidate genes screened by CNVs could be used as relevant molecular markers for the improvement of Tibetan sheep breeds.

Selective sweeps can reveal putative regions under environmental and artificial selection during local adaptation and domestication [60]. In the genome, calculating paired V_ST_ values can be used to screen for key CNVRs that differ significantly between populations [43]. We identified a total of 368 differential CNVRs between the two breeds and further identified 41 functional genes located within these CNVRs. Through annotation analysis, it was found that these genes were enriched in aspartic acid metabolism, kinase binding, ATPase-coupled transmembrane transporter activity regulation and other processes and participated in multiple metabolic pathways. In the present study, five CNVRs carrying the *XIRP2*, *ABCB1*, *CA1*, *ASPA* and *EEF2* genes showed significant pairwise differentiation between Oula and Panou sheep. The *XIRP2* gene has been reported to be associated with feed efficiency, average daily gain and residual feed intake in cattle [61]. Subsequently, Seifi Moroudi et al. also demonstrated that the *XIRP2* gene was associated with growth and developmental biology pathways in sheep and cattle [62]. This function of the *XIRP2* gene is important for Tibetan sheep living in a relatively pasture-deficient area. A previous study found that the *ABCB1* gene was hypoxia-responsive and could be regulated by HIF-1α due to a hypoxia-responsive element on its promoter [63]. Moreover, in the gut of sheep, the relative expression of the *ABCB1* gene in different compartments differed greatly, and there was a certain gradient, which indicates that it plays an important role in the digestive metabolic process in sheep [64]. Berton et al. found that the *ABCB1* gene was also involved in the gastrointestinal immune response and inflammatory processes in sheep [65]. Sales et al. explored the relationship between the *ABCB1* gene and folliculogenesis by vitrification and in vitro culture of sheep ovarian tissue, indicating that the *ABCB1* gene may affect the development of ewe follicles [66]. Thus, it seems that the *ABCB1* gene plays an important role in the adaptation of Tibetan sheep to the plateau environment. Carbonic anhydrase 1 (*CA1*) is an enzyme abundant in colonic epithelial cells and plays a role in the maintenance of cellular acid-base homeostasis [67]. *CA1* is associated with many physiological and pathological processes, including the development of various digestive disorders, gluconeogenesis and adipogenesis. In addition, polymorphic loci in the *CA1* gene could be used as potential genetic markers for improving feed efficiency in sheep [68]. The *CA1* gene is important for Tibetan sheep living at high altitudes to maintain their growth and metabolic balance, enhance disease resistance and improve feed utilization. Melatonin secretion is closely related to oestrous in sheep, and the *EEF2* gene induces melatonin secretion from the pituitary gland to participate in the oestrous of sheep [69]. Zhang et al. found that the relative expression of the *ASPA* gene in the hypothalamus changed significantly during oestrous and was associated with litter size in sheep [70]. During domestication, several genes associated with the physical characteristics of sheep were artificially selected in a targeted manner. Therefore, under these selection pressures, CNVs may accumulate in sheep breeds, thus forming the genetic basis of important economic characteristics. In this study, the Panou sheep is a cultivated breed with the local Oula Tibetan sheep as the hybrid female and its wild-line ancestor, the Argali sheep, as the hybrid male, after a long period of adaptation to the low oxygen environment of the plateau and artificial selection. Therefore, we believe that the CNVRs of Panou sheep carry five genes, *XIRP2*, *ABCB1*, *CA1*, *ASPA* and *EEF2*, which are also subject to strict selection pressure during breed formation. This may help them better adapt to the low oxygen and cold environment in high-altitude areas and the lack of growth conditions for pasture, which is in line with our initial breeding goals.

## Conclusions

In this study, based on whole genome resequencing of 20 Tibetan sheep, we explored the genomic characteristics and structural variation of two breeds of Tibetan sheep and 368 CNVRs were obtained. By analysing the functions of the annotated genes in the CNVRs and the genes showing copy number differences among varieties, we found that the genetic variation caused by artificial selection was effective at the CNV level. Some CNVRs carry differential genes, such as *XIRP2*, *ABCB1*, *CA1*, *ASPA* and *EEF2*, that may be related to body weight, environmental adaptation and reproductive capacity. Our research results provide valuable resources of the genome-wide variation for Tibetan sheep and are useful for elucidating the genetic basis of plateau adaptation in Tibetan sheep. Furthermore, these results will contribute greatly to the future breeding and development of economic traits in Tibetan sheep.

### Electronic supplementary material

Below is the link to the electronic supplementary material.

Supplementary Material 1

Supplementary Material 2

Supplementary Material 3

Supplementary Material 4

Supplementary Material 5

Supplementary Material 6

Supplementary Material 7

Supplementary Material 8

Supplementary Material 9

Supplementary Material 10

## Data Availability

The datasets generated in this paper can be found at Sequence Read Archive: PRJNA797957. Link address: https://www.ncbi.nlm.nih.gov/bioproject/PRJNA797957.
